# Evaluation of Cytotoxicity of 4-Hydroxycinnamic Acid Using Tetrazolium Bromide Assay and Zebrafish Embryotoxicity: An In-Vitro Study

**DOI:** 10.7759/cureus.55915

**Published:** 2024-03-10

**Authors:** Siddhant U Thorat, Ravindra Kumar Jain, Karthikeyan Ramalingam, Saheb Ali, Shankar Ganesh

**Affiliations:** 1 Orthodontics and Dentofacial Orthopaedics, Saveetha Dental College and Hospitals, Saveetha Institute of Medical and Technical Sciences, Saveetha University, Chennai, IND; 2 Oral Pathology and Microbiology, Saveetha Dental College and Hospitals, Saveetha Institute of Medical and Technical Sciences, Saveetha University, Chennai, IND; 3 Periodontics, Saveetha Dental College and Hospitals, Saveetha Institute of Medical and Technical Sciences, Saveetha University, Chennai, IND; 4 Centre for Infectious Diseases, Department of Microbiology, Saveetha Dental College and Hospitals, Saveetha Institute of Medical and Technical Sciences, Saveetha University, Chennai, IND

**Keywords:** zebrafish cytotoxicity, mtt assay, in vitro cytotoxicity, cytotoxicity, antimicrobial, 4-hydroxycinnamic acid

## Abstract

Aim

This study aimed to evaluate the cytotoxicity of a novel compound, 4-hydroxycinnamic acid (4-HCA), with the help of a 3-(4,5-dimethylthiazol-2-yl)-2,5-diphenyltetrazolium bromide (MTT) assay and zebrafish embryotoxicity.

Materials and methods

In this in vitro study, MTT fibroblast assays using dental pulp stem cells, which were cultured in Modified Eagle’s Medium or Dulbecco's Modified Eagle Medium, and zebrafish cytotoxicity and embryotoxicity were done to evaluate the cytotoxicity of the novel compound 4-HCA. The data was analyzed by plotting cell number versus absorbance, allowing quantitation of changes in cell proliferation.

Results

4-HCA (40 μl) showed acceptable levels of cell viability according to the American Society for Testing and Materials standards. Cell viability is reduced with increased exposure time and concentrations of 4-HCA. Similarly, the cytotoxicity assessment in zebrafish (*Danio rerio*) showed an acceptable range of toxicity levels in embryonic stages used to evaluate the mortality rate of zebrafish embryos.

Conclusion

Considering the constraints of this research, it can be deduced that hydroxycinnamic acid at a concentration of 40 μl was non-toxic. The findings from the MTT assay indicated a correlation between the concentration and the toxicity of the compound. Likewise, the zebrafish test demonstrated minimal toxicological effects.

## Introduction

4-hydroxycinnamic acid (4-HCA), a hydroxy derivative of cinnamic acid, also known as p-coumaric acid, is a naturally occurring organic compound that belongs to a class of phenolic compounds. Found in various plants, including fruits, vegetables, and grains, it plays a role in plant metabolism and is considered for its phytochemicals with potential health benefits [1]. The latest research related to HCA bioavailability and their biological effects revealed by epidemiological data and pre-clinical and clinical studies shows the beneficial effects of HCA in patients with CVD, obesity, diabetes, brain disorders, cancers, and neurodegenerative diseases [2].

Some studies have reported on the anti-tumor and antioxidant properties of 4-HCA [3,4]. The selective and highly potent activity of 4-HCA against resistant biofilms of gram-positive medical device-related pathogens has been reported [5]. Activity against biofilms of *Staphylococcus aureus* and *Escherichia coli* were reported in a study by Laverty et al. [5]. In a recent study by Ramsundar et al., the anti-quorum sensing and anti-biofilm activity of 4-HCA acid against *Streptococcus mutans* (*S. mutans*) isolated from subjects undergoing orthodontic treatment showed remarkable results. The growth of *S. mutans* was inhibited at remarkably low concentrations, starting at 0.31 mg/ml. Consequently, 4-HCA demonstrates potential as a powerful antimicrobial agent against *S. mutans*, suggesting its potential incorporation into oral prophylactic devices pending further testing [6]. Due to their health-related applications in cosmetics, dentistry, and medicine owing to their anti-inflammatory, anti-oxidant, and cytoprotective properties, HCAs hold significant value.

Previously, cytotoxicity was assessed for HCA in tumor cell lines by Kozubek et al. [3]. The cytotoxicity of various novel compounds can be evaluated using different methods. These methods include human gingival fibroblast assay, 3-(4,5-dimethylthiazol-2-yl)-2,5-diphenyltetrazolium bromide (MTT) fibroblast assay, brine shrimp lethality test, and zebrafish (*Danio rerio*) embryo cytotoxicity [7-9].

As 4-HCA is a relatively novel compound, very little literature exists on the cytotoxicity of this compound and its related compounds. Hence, the present study aimed to assess the potential cytotoxic effects of the newly synthesized compound 4-HCA with an MTT fibroblast assay utilizing dental pulp stem cells (DPSC). These cells were cultured in Eagle’s medium or Dulbecco's Modified Eagle Medium (DMEM). Additionally, zebrafish cytotoxicity and embryotoxicity tests were carried out.

## Materials and methods

This in-vitro study was conducted in a university setting at the Blue Lab of the Research Department at Saveetha Institute of Medical and Technical Sciences, Chennai (SRB/SDC/ORTHO-2202/23/095). The novel compound 4-HCA was tested for its in vitro cytotoxicity in this study using an MTT assay of DPSC and zebrafish embryos.

Collection of synthetic compound

4-HCA was purchased from Sigma-Aldrich (St. Louis, MO) in the form of a powder.

Cytotoxicity evaluation

MTT Assay

**Figure 1:**
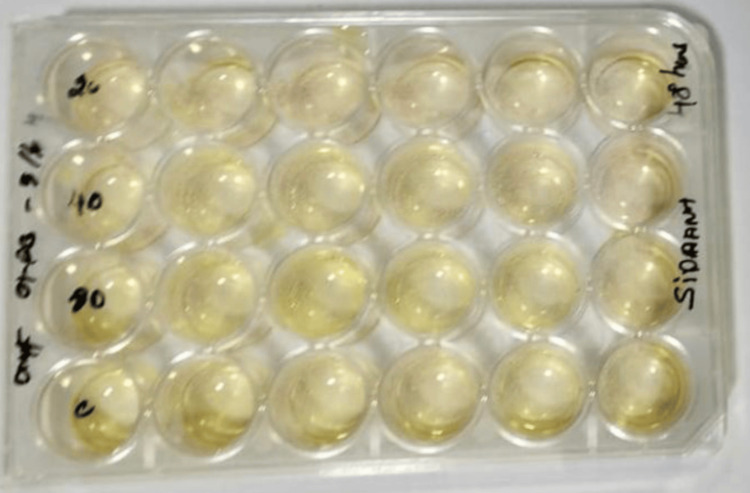
Dental pulp stem cell (DPSC) seeded wells

**Figure 2:**
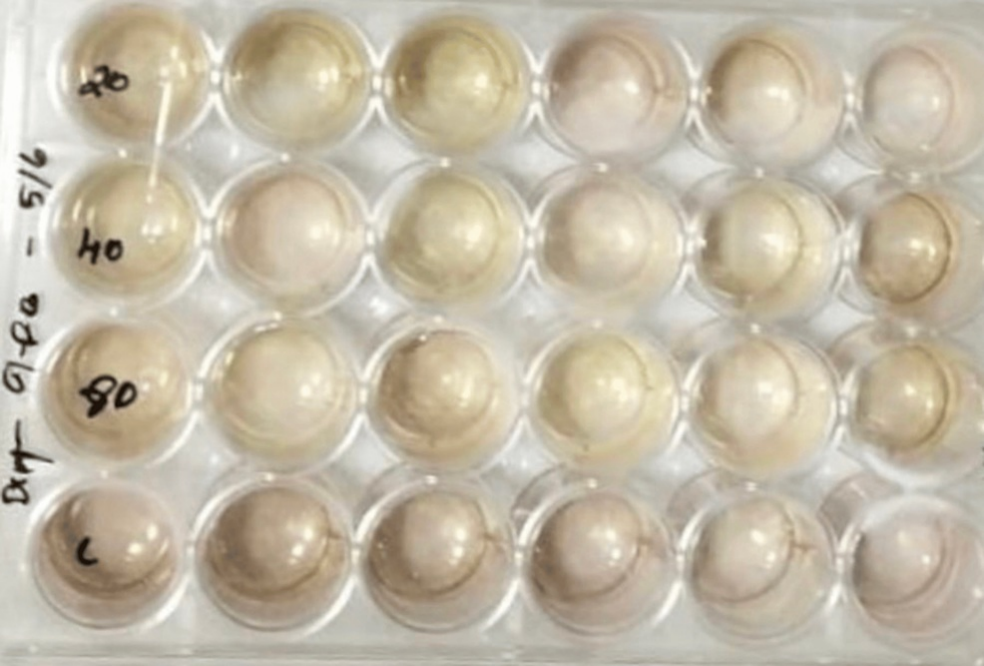
Purple color changes due to the formation of formazan crystals

The MTT assay is a commonly used colorimetric assay that measures cell metabolic activity and is often employed to assess cell viability, proliferation, and cytotoxicity. DPSCs have been proposed as an alternative to pluripotent stem cells to study multilineage differentiation in vitro and for therapeutic applications. DPSCs have high proliferation rates, are clonogenic, and possess all the properties of stem cells. They can differentiate into dental pulp tissue as well as pulp complexes and can probably be used to treat injured or infected pulp tissues. These cells were cultured in Modified Eagle minimum essential medium F12 with 15% (vol/vol) heat-inactivated FBS, 2 mM L-glutamine, 50 IU/mL penicillin, and 50 mg/mL streptomycin. After one week, the cells were dissociated using a trypsin solution and then replated in six-well plates at a cell density of 2.5 x 10^5 cells per well. Two milliliters of DMEM were added to each well after 24 hours of cell attachment in the six-well plate with 1 ml of complete culture medium per well. Subsequently, 0.5 mg/ml MTT was introduced into the lower well, and the plate was incubated at 37°C for four hours. Following the incubation period, the culture medium was removed from both the insert and well, and the formazan crystals obtained were dissolved by adding 100 µl of dimethyl sulfoxide (DMSO) solution to each well. The cell types were delicately agitated for two minutes to ensure even mixing with the blue reaction products in the solvent. Subsequently, 100 µl of the colored DMSO was transferred from each insert and well to a fresh 96-well plate for the quantification of cell viability. The microplate reader was employed to measure absorbance at 450 nm [10,11]. A higher absorbance indicates a larger population of viable and metabolically active cells in the solution (Figures 1-2).

Zebrafish cytotoxicity study

Zebrafish (*Danio rerio*) embryos were used in this study to evaluate the in vivo embryonic toxic and cytotoxic assessment on organogenesis and wound healing. Zebrafish of Indian-bred origin cultured in the Green Lab, Research Department, Saveetha Dental College, and hospitals were maintained and bred in the laboratory at room temperature for over two years (over four generations). Samples from both genders were subjected to the HCA compound following the induction of an artificial injury near the zebrafish's caudal fin using a soft tissue laser, an Epic 940 nm diode laser (US Biolase).

Zebrafish embryotoxicity assay

4-HCA at a concentration of 40 μl was exposed to zebrafish and was used to evaluate the mortality rate of zebrafish embryos. The evaluation of 4-HCA in vitro cytotoxicity was conducted by examining zebrafish embryos. The evaluation of the mortality rate in zebrafish after exposure to 4-HCA was done at fixed time points and in comparison with untreated embryos (control). The toxicity was assessed on a group of twenty chosen zebrafish embryos exposed to a concentration of 40 μl in Hank's solution. Subsequently, the eggs were transferred to individual wells to facilitate the development of the eyes, head, and tail, which were then examined under a 40X microscope (Olympus PLN 40X Objective) at 24-hour intervals. During the assessment, a constant normal room temperature was maintained with respect to the solution. The death rate of hatched embryos was evaluated every 24 hours for five days [12-14].

Tissue regeneration in zebrafish

The artificial injury created in the caudal part of the zebrafish was exposed to the compound at three different concentrations (20 μl, 40 μl, and 80 μl) while it simultaneously underwent healing and regeneration. A thin section of tissue undergoing wound healing was examined under a light microscope after it was stained with hematoxylin and eosin. Following tissue fixation, dehydration, and embedding in paraffin, thin sections were cut using a Semi-Automated Rotary Microtome M-240 (Myr, Spain) and mounted on glass slides. The sections were stained with hematoxylin, a basic dye that imparts a blue or purple color to acidic structures such as cell nuclei, and eosin, an acidic dye that stains cytoplasm and other basic components in shades of pink or red. After staining, the sections were dehydrated, cleared, and coverslipped. The resulting H&E-stained slides were examined under a microscope. The sections were observed and documented for wound healing features such as inflammation, cell proliferation, and tissue remodeling. Images of the same were captured for documentation and analysis of tissue regeneration in zebrafish.

## Results

MTT assay

24-Hour Cell Viability 

**Figure 3:**
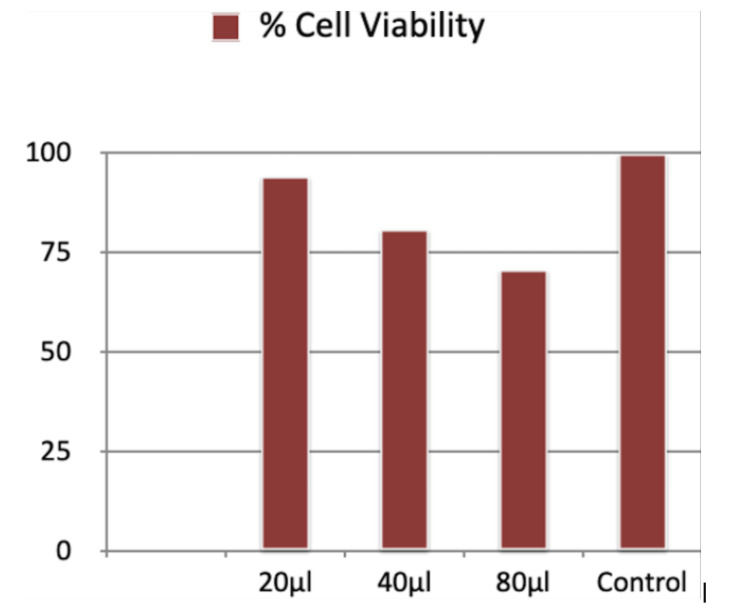
Percent cell viability for 24-hour cell viability

Table 1 and Figure 3 give the results of the 24-hour MTT assay at HCA concentrations of 20 μl, 40 μl, and 80 μl. The cell viability was 93.95%, 80.64%, and 70.69% in comparison to the control, which had a cell viability of 100%. The results indicated that with an increase in the concentration of HCA, there was a progressive fall in the percentage of cell viability.

**Table 1:** Results for 24-hour cell viability for MTT assay

	Average	Group average	Standard deviation	% cell viability
Concentration-20	0.17	0.14	0.02	93.95
Concentration-40	0.12	0.13	0.01	80.64
Concentration-80	0.11	0.13	0.01	70.69
Control	0.15	0.15	0.01	100

**Figure 4:**
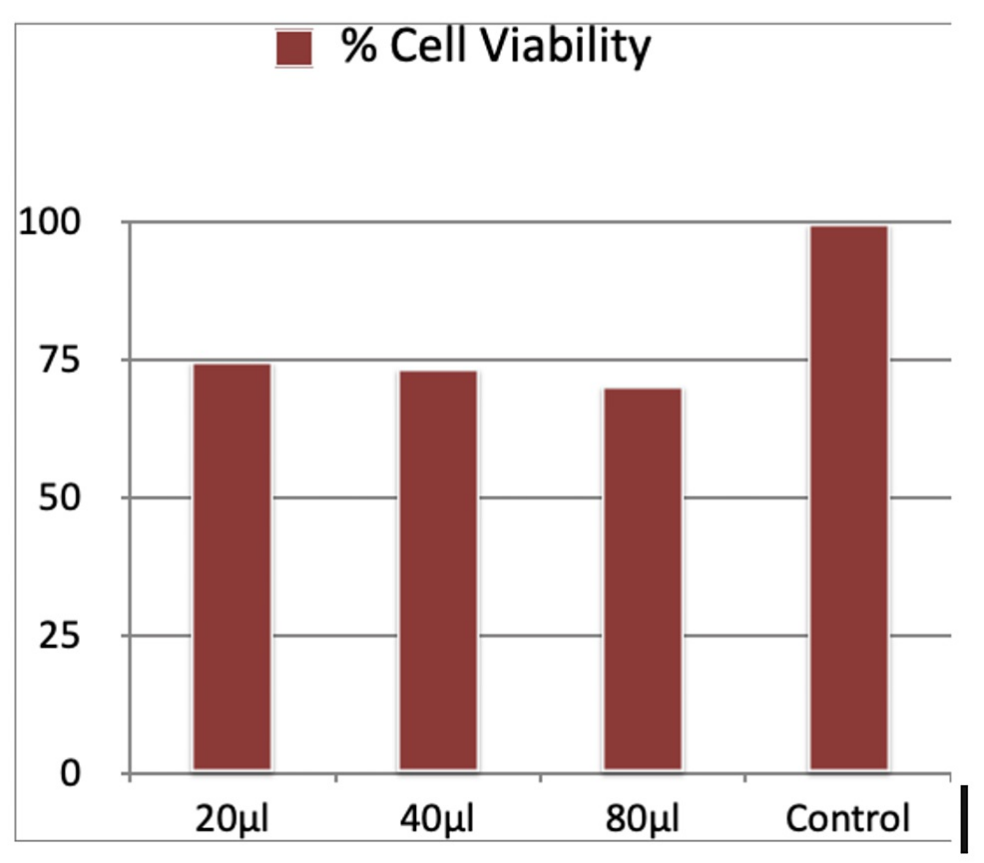
Percent cell viability for 48-hour cell viability

Table 2 and Figure 4 give the results of the 48-hour MTT assay at HCA concentrations of 20 μl, 40 μl, and 80 μl. The cell viability was 74.61%, 73.44%, and 70.41% in comparison to the control, which had a cell viability of 100%. The results of the 48-hour MTT assay also indicated that with an increase in the concentration of HCA, the cell viability decreased gradually.

**Table 2:** Results for 48-hour cell viability for MTT assay

	Average	Group average	Standard deviation	% cell viability
Concentration-20	0.16	0.17	0.06	74.61
Concentration-40	0.16	0.18	0.01	73.44
Concentration-80	0.15	0.19	0.02	70.41
Control	0.22	0.22	0.02	100

48-Hour Cell Viability

**Figure 5:**
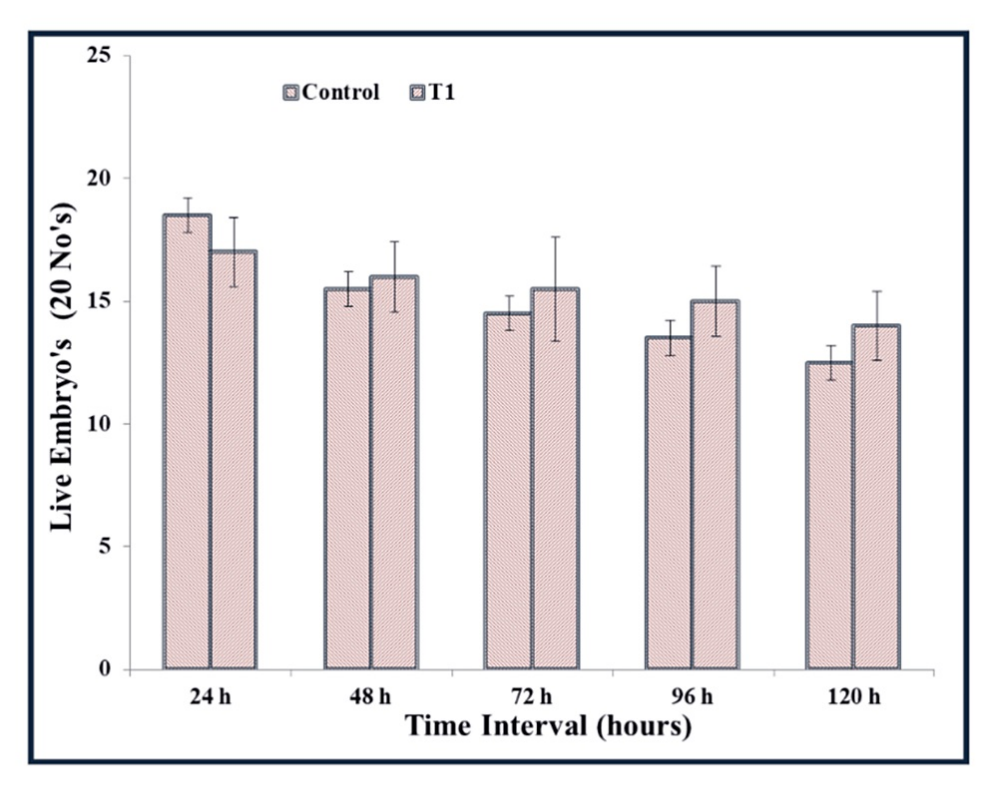
Bar chart showing the number of dead and live zebrafish embryos at different time intervals after exposing to 40 μg/mL of HCA (T1 - test sample 1) and in the control group

**Figure 6:**
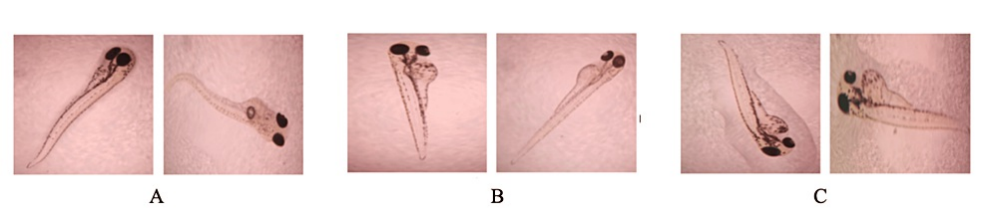
Zebrafish embryo cytotoxicity assay A: Microscopic slides showing zebrafish embryos in control groups. B: After 96 hours of being exposed to 40 μg/mL. C: 120 hours of being exposed to 40 μg/mL

**Figure 7:**
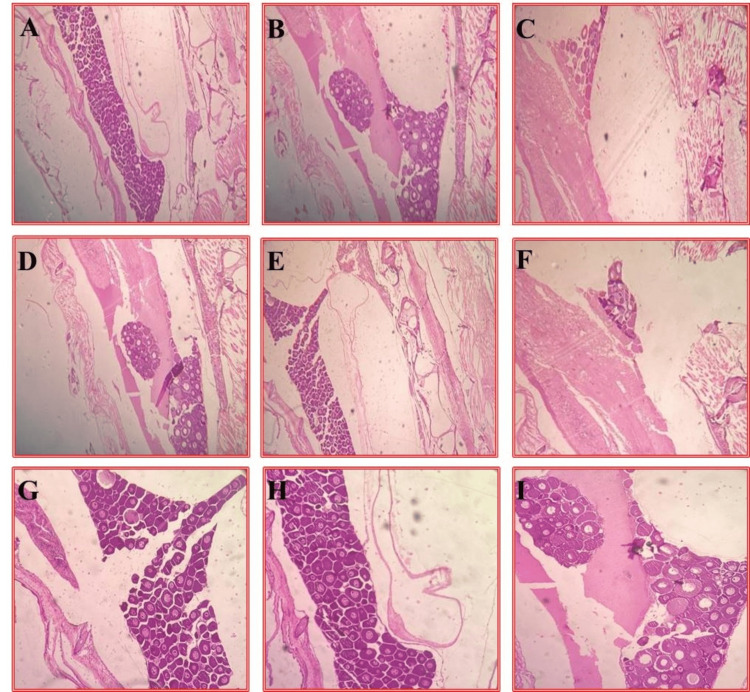
H&E stained sections of zebrafish showing regeneration of tissue followed by treatment with 4-HCA treatment with a standard control A-C: Control, low concentration 20 mg/mL 40 X observation. D-F: Control, high concentration 40 mg/mL with 40 X observation. G-I: control, LC and HC with 4 X concentration Compared to control, epithelial cell deposition indicates regeneration potential with the treatment of low-concentration material. On the other hand, with the incubation of high-concentration material, the growth of epithelial cells was diminished. Therefore, it can be understood that at higher concentrations, due to the toxicology effect, the growth of epithelial cells may be compromised

Zebrafish embryo cytotoxicity assay: For cytotoxicity assessment, 40 μl of 4-HCA was dispersed in Hank’s solution. The zebrafish embryos were screened for toxicity based on the number of embryos that were live or dead after treatment with 4-HCA. Using a light microscope under 40x magnification, the 4-HCA-treated embryos and mature eggs in 24 and 48 hours were observed. The 4-HCA obtained displayed a mortality rate of 0.70 ± 2.12% at a concentration of 40 μg/mL. At the 72-hour mark, the developed eggs were examined using a microscope to identify the formation and deformities of the tail, head, and eyes when exposed to 4-HCA. Furthermore, at 92 and 120 hours, the majority of the embryos had hatched after treatment. Delayed hatching in the prior stage of treatment was noted for some of the 4-HCA-treated embryos. The obtained results concluded that at a concentration of 40 μg/mL, 4-HCA showed low cytotoxicity as earlier embryonic developmental stages showed delayed hatching time. The delay in the hatching process might result from the embryo’s adaptation for hatching, but it does not indicate any toxic attributes. The results of zebrafish embryotoxicity showed very low cytotoxicity values. Hence, 4-HCA can be used for applications in biomedicine due to its good biocompatibility (Figures 5-7).

## Discussion

The present study aimed to evaluate the cytotoxicity of the novel compound 4-HCA with the help of an MTT assay and zebrafish embryotoxicity. HCAs, along with polyphenols in general, represent phytochemicals that offer numerous advantages for the health of plants, animals, and humans [15]. HCAs are typically not available to humans in their free form, where they appear to exert their primary positive impacts, but are instead linked to intricate polysaccharides within plant cell wall structures (dietary fibers) [16]. 4-HCA has strong antibacterial activity, which can be attributed to the presence of a hydroxy or a methoxy group in the HCA conjugates, which show a higher radical-scavenging activity than the phenol and ester derivatives [3]. A previous study evaluating the cytotoxic properties of HCA anticipated a positive correlation between cytotoxic and antioxidant activities, inherently linked to physico-chemical and conformational properties. Subsequent research will determine whether modifying the substitution pattern of the cinnamic acid core can enhance both cytotoxic effects and selectivity [3].

A study done by Ramsundar et al. revealed the minimum inhibitory concentration of 4-HCA to be 40 μl [6]. The present study revealed that the novel compound, 4-HCA, is non-toxic in vitro when used at the recommended concentration of 40 μl. The compound had less cytotoxicity, which was within the acceptable range according to the American Society for Testing and Materials (ASTM) standards [17]. The cytotoxicity test is one of the most important indicators of the biological evaluation system in vitro, and with the progress of modern cell biology, experimental methods to evaluate cytotoxicity are also continuously being developed and improved. Assay methods, grounded in diverse cell functions like enzyme activity, cell membrane permeability, adherence, ATP and co-enzyme production, and nucleotide uptake, encompass a range of techniques [18]. These methods can be broadly categorized into: (1) dye exclusion approaches like the trypan blue dye exclusion assay; (2) methods reliant on metabolic activity; (3) ATP assay; (4) sulforhodamine B assay; (5) protease viability marker assay; (6) clonogenic cell survival assay; (7) DNA synthesis cell proliferation assays; and (8) Raman microspectroscopy [19]. To select the most suitable viability assay, careful consideration of cell type, culture conditions, and specific research questions is essential.

The results of this study showed that the compound 4-HCA (40 μl) showed acceptable levels of cell viability according to the ASTM standards when studied with DPSC in an MTT assay. The study results indicate that with an increase in exposure time to HCA, there was a decrease in cell viability of 80.64% and 73.44% at 24 and 48 hours, respectively, for the concentration of 40 μl. Similarly, the cytotoxicity assessment in zebrafish (*Danio rerio*) showed an acceptable range of toxicity levels in embryonic stages.

Similar studies have been performed in the past to assess the cytotoxicity of HCA. In a study by Kozubek et al., four different types of HCA were evaluated for their cytotoxicity against different human tumor cell lines [3]. The results of this study are in line with the results of the present study, as all the varieties of HCA exhibited low cytotoxicity.

However, it is important to consider the limitations of this study. This study was a preliminary study performed in vitro. In the future, studies evaluating the cytotoxicity of acid at various concentrations and under simulated clinical conditions could be carried out in order to further substantiate the non-toxic nature of this compound.

## Conclusions

The in vitro and in vivo cytotoxicity tests for 4-HCA showed favorable results. The results of the MTT assay revealed that the toxicity of this compound increased with an increase in concentration. In relatively low concentrations, the compound shows good biocompatibility. Similarly, the results of the zebrafish test exhibited low toxicology results. Hence, 4-HCA can be used for applications in biomedicine due to its good biocompatibility. The fact that this study is conducted in vitro represents a limitation. Therefore, it's imperative for the synthetic compound to undergo further assessment, which should involve experimental animals. Subsequently, human clinical trials must be initiated. Future prospects entail a comprehensive evaluation of the compound and an investigation of its potential application as an active agent for maintaining oral hygiene in individuals undergoing orthodontic treatment.
